# Pseudokinase NRP1 facilitates endocytosis of transferrin in the African trypanosome

**DOI:** 10.1038/s41598-022-22054-x

**Published:** 2022-11-03

**Authors:** Gaurav Kumar, Bryanna Thomas, Kojo Mensa-Wilmot

**Affiliations:** 1 Department of Molecular and Cellular Biology, Kennesaw State University, Kennesaw, Georgia; 2 Center for Tropical and Emerging Global Diseases, University of Georgia, Athens, Georgia

**Keywords:** Parasite biology, Parasite physiology

## Abstract

*Trypanosoma brucei* causes human African trypanosomiasis (HAT) and *nagana* in cattle. During infection of a vertebrate, endocytosis of host transferrin (Tf) is important for viability of the parasite. The majority of proteins involved in trypanosome endocytosis of Tf are unknown. Here we identify pseudokinase NRP1 (Tb427tmp.160.4770) as a regulator of Tf endocytosis. Genetic knockdown of NRP1 inhibited endocytosis of Tf without blocking uptake of bovine serum albumin. Binding of Tf to the flagellar pocket was not affected by knockdown of NRP1. However the quantity of Tf per endosome dropped significantly, consistent with NRP1 promoting robust capture and/or retention of Tf in vesicles. NRP1 is involved in motility of Tf-laden vesicles since distances between endosomes and the kinetoplast were reduced after knockdown of the gene. In search of possible mediators of NRP1 modulation of Tf endocytosis, the gene was knocked down and the phosphoproteome analyzed. Phosphorylation of protein kinases forkhead, NEK6, and MAPK10 was altered, in addition to EpsinR, synaptobrevin and other vesicle-associated proteins predicted to be involved in endocytosis. These candidate proteins may link NRP1 functionally either to protein kinases or to vesicle-associated proteins.

## Introduction

*Trypanosoma brucei* causes human African trypanosomiasis (HAT) and the cattle disease *nagana* in regions of rural sub-Saharan Africa^1^. The majority of genes in *T. brucei* are annotated as “hypothetical” (i.e., proteins with unknown function) in genome databases^2^ due to extensive divergence of protein sequences from those of model eukaryotes. Consequently bioinformatic methods to predict protein function (e.g., sequence alignments) identifies a partial list of potential members of biological pathways in a trypanosome^3^. Other approaches are needed to complement bioinformatic analysis in order to fully characterize biological pathways in trypanosomatids.

Endocytosis of host transferrin (Tf) is essential for viability of bloodstream *T. brucei* in a vertebrate^4,5^. In human cells, the pathway for Tf endocytosis is well-characterized (reviewed in^6^). There, Tf is endocytosed through clathrin-coated vesicles after binding to a transmembrane receptor (TfR)^7,8^. Cytoplasmic regions of TfR interacts with adaptor protein-2 (AP-2)^9^ which also binds PIP_2_ (phosphatidylinositol 4,5-bisphosphate) at the plasma membrane^10,11^ to facilitate assembly of a clathrin (vesicle coat protein) lattice^12^. Clathrin-coated vesicles bud from the plasma membrane with assistance of a GTPase dynamin^13^, and the internalized vesicles fuse to form tubular transport endosomes that, assisted by motor proteins, move along microtubule tracks^14^. The trypanosome Tf endocytosis system differs significantly from that of humans (reviewed in^15,16^). First, a trypanosome Tf receptor (TbTfR) is not a transmembrane protein; it is a heterodimer of ESAG6/ESAG7 that is anchored to the plasma membrane by a glycophosphatidylinositol (GPI) anchor (reviewed in^17^). Thus, TbTfR lacks a cytoplasmic domain that would recruit cytoplasmic adapter and coat proteins for vesicle biogenesis. Second, *T. brucei* lacks AP-2^16^ which in humans is essential for endocytosis of Tf^18^. It appears that EpsinR might be an adaptor for uptake of Tf^19^. Third, knockdown of a dynamin homologue in *T. brucei* has no effect on Tf endocytosis^20^. These differences underscore a need for new approaches, besides protein sequence alignments, to identify proteins involved in trypanosome Tf endocytosis.

Protein phosphorylation regulates Tf endocytosis in human cells^21–24^. In *T. brucei* Tf endocytosis is reduced by Tyrphostin A47, a pan-inhibitor of tyrosine kinases^25^. Further, knockdown of glycogen synthase kinase-3β (TbGSK3β) blocks Tf endocytosis^26^.

Small-molecules can be used as tools to perturb biological pathways^27–30^. Analysis of proteins altered by small-molecules may reveal novel factors associated with biological pathways that are perturbed by the small-molecules^31–33^. Thereafter, genetic approaches can be used to validate an identified protein’s role in the process, permitting discovery of new “pathway proteins” without reliance on alignment of protein sequences.

The small molecule AEE788^34^ inhibits Tf endocytosis in *T. brucei*^35^, and was used as a perturbant of the pathway. Analysis of the phosphoproteome of *T. brucei* after treatment with AEE788 revealed possible mediators of endocytosis^35^. Tb427tmp.160.4770 (protein ID^2^) was hyper-phosphorylated at the same time that Tf endocytosis was inhibited 90%^35^. Tb427tmp.160.4770 is 30% identical in the kinase domain to that of AP-2 Associated Kinase 1, a regulator of clathrin-mediated endocytosis in human cells^36,37^, and a member of the Numb-associated family of protein kinases (NAKs). We refer to Tb427tmp.160.4770 as NAK Related Pseudokinase (NRP1), because it lacks HRD and DFG motifs required for catalysis by protein kinases^38–40^.

Here we establish NRP1’s importance in selective endocytosis of Tf. Intracellularly, endocytosed Tf colocalizes with NRP1 as well the vesicle protein clathrin. From genetic knockdown experiments, we surmise that NRP1 (1) does not affect binding of Tf at the flagellar pocket; (2) controls accumulation of Tf in endosomes, and (3) NRP modulates motility of endosomes. NRP1 is a new member of the set of proteins involved in the endocytosis Tf in the African trypanosome.

## Results

### Discovery of an endocytosis pathway protein using AEE788 perturbation proteomics in a trypanosome

AEE788^34^ inhibits transferrin (Tf) endocytosis in *T. brucei* in a time-dependent fashion^35^. After four hours of treatment with AEE788 no inhibition of transferrin (Tf) endocytosis was observed. However, after a 9-h exposure AEE788 inhibited Tf endocytosis^35^. In contrast, bovine serum albumin (BSA) endocytosis increased, and tomato lectin uptake was not affected at the 9-h time point. These data hinted at the potential utility of AEE788, which can complex with trypanosome protein kinases^41^, as a small-molecule perturbant of Tf endocytosis pathways in *T. brucei*. Comparative analysis of the drug’s effect on the phosphoproteomes at 4 h and 9 h revealed that Tb427tmp.160.4770 was hyper-phosphorylated only in trypanosomes treated for 9 h with AEE788^35^.

**Figure 1:**
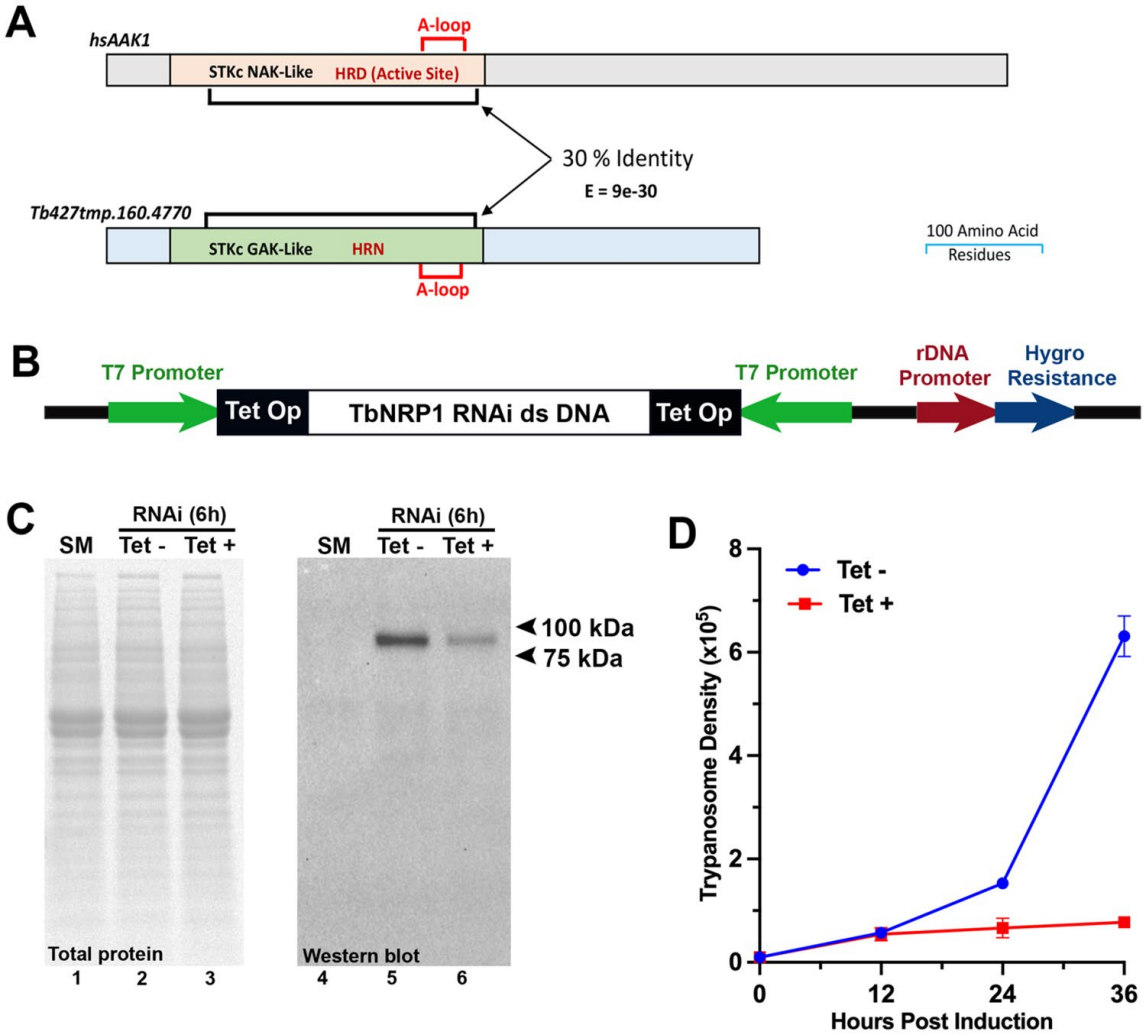
RNAi knockdown of TbNRP1. (**A**) Domains of TbNRP1 and human AAK1 (hs AAK1) (UniProtKB: Q2M2I8). Sequence alignment was carried out on NCBI Protein Blast. Feature and domain annotation were determined from NCBI Conserved Domain search. Black brackets show 30% sequence identity between kinase and kinase-like domain of hsAAK1 and TbNRP1. (**B**) Cartoon of p2T7^TABlue^ derived RNAi construct. Dual opposing T7 promoters drive expression of a TbNRP1 fragment under tetracycline regulation. Hygromycin resistance expression is driven constitutively a rDNA promoter. (**C**) TbNRP1 protein level 6 h after induction of knockdown. Trypanosomes (2 × 10^6^) were lysed, and proteins were separated by SDS-PAGE (12%). Protein was transferred to a PVDF membrane and developed using anti-V5 antibody. Stain-Free blot shows total protein load. (**D**) Trypanosome proliferation after knockdown of TbNRP1. Cells were seeded at 1 × 10^4^ cells/ mL, knockdown was induced with tetracycline (1 µg/mL) and cell density determined with a hemocytometer every 12 h for 36 h. Error bars represent standard deviation from three biological samples.

Tb427tmp.160.4770 (named TbNRP1 in this manuscript) is a pseudokinase. A canonical HRD motif found in enzymatically functional protein kinases (reviewed in^42^) is absent in TbNRP1, replaced with an HRN motif present in other pseudokinases^43^. TbNRP1 also lacks a DFG motif that is involved in magnesium binding during catalysis^39^: The protein has 30% identity (Fig. 1A) with the kinase domain of Human AP2-associated kinase 1 (HsAAK1) that regulates endocytosis^37^.

To assess possible importance of TbNRP1 in trypanosome biology, a single marker (SM) bloodstream line^44^ was stably transfected with an RNAi construct containing a fragment of TbNRP1 (Fig. 1B)^45^. To knock down TbNRP1, tetracycline (1 µg/mL) was added to trypanosomes for 6 h, and loss of TbNRP1 protein was monitored by western blotting (Fig. 1C). TbNRP1 protein was reduced 62% (std dev ± 3.7, n = 3) at 6 h (after normalizing for total protein load). Density of proliferating trypanosomes was measured every 12 h for 36 h (Fig. 1D). After 6 h of knockdown, the number of kinetoplasts (K) and nuclei (N) per trypanosome (6 h) was not significantly affected (Supp. Fig. S1). At 14 h proliferation ceased in Tet-induced trpanosomes (Fig. 1D).

**Figure 2:**
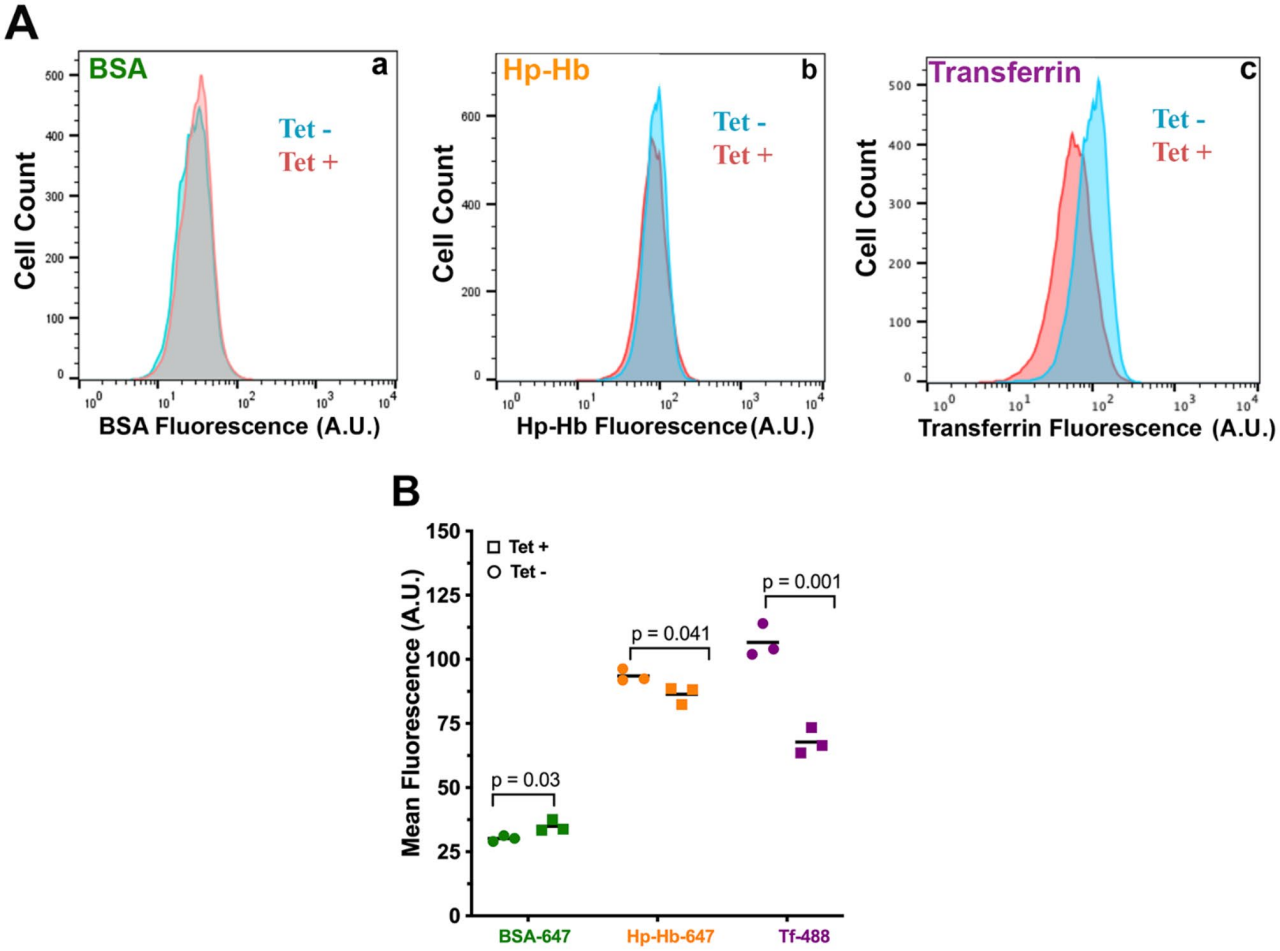
TbNRP1 selectively regulates transferrin endocytosis. Knockdown of NRP1 was induced for 6 h with 1 µg/mL tetracycline. Cells were incubated with BSA-Alexa647 (25 µg/mL), Hp-Hb-Alexa647 (30 µg/mL) or Transferrin-Alexa488 (25 µg/mL) for 15 min at 37 °C. Fluorescence was analysed with flow cytometry. (**A**) Representative histograms of fluorescence intensity in uninduced (Tet-) and induced (Tet+) cells for BSA-Alexa647, Hp-Hb-Alexa647 and Transferrin-Alexa488, respectively. (**B**) Quantitation of mean fluorescence of BSA-Alexa647, Hp-Hb-Alexa647 and Tf-Alexa488 in uninduced (Tet-) and induced (Tet +) cells (n = 3). *p* value was calculated using Unpaired Student’s t-test. Bars represent mean values.

To determine whether (or not) TbNRP1 affected endocytosis, uptake of different protein cargo (Tf, BSA, and haptoglobin/haemoglobin (HpHb)) was studied using flow cytometry (Fig. 2A). Endocytosis of Tf (Tf-Alexa488) decreased by 38% after 6 h of TbNRP1 knockdown (*p* = 0.001, n = 3) (Fig. 2B), and uptake of HpHb-Alexa488 was reduced by 8% (*p* = 0.04, n = 3). In contrast, endocytosis of BSA increased by 10% after knockdown of TbNRP1 (*p* = 0.03, n = 3) (Fig. 2B). Based on these results, we conclude that TbNRP1 is important for efficient Tf endocytosis in the African trypanosome.

### Binding of Tf at the flagellar pocket is not affected by knockdown of TbNRP1

Endocytosis of Tf is initiated at the flagellar pocket of *T. brucei*^46^. Since knockdown of TbNRP1 reduces endocytosis of Tf (Fig. 2) we sought to determine the defective step in the uptake of Tf by testing whether (1) binding of Tf to its receptor at the flagellar pocket or/and (2) internalization of Tf was reduced after knockdown of TbNRP1. We used the two temperatures to investigate different aspect of endocytosis. At low temperature (3 °C), endocytosis of Tf is inhibited and receptor-bound Tf is detected at the flagellar pocket^47^. At 37 °C, scission of Tf vesicles from the plasma membrane takes place from the flagellar pocket and endosomes are found between kinetolast and nucleus; the endosomes are en route to the lysosome that is located near the nucleus.

Flagellar pocket binding of Tf-AF594 was monitored before and after knockdown of TbNRP1 (Fig. 1C). At low temperature (3 °C), endocytosis of Tf is inhibited and receptor-bound Tf is detected at the flagellar pocket^47^. Transgenic trypanosomes (Fig. 1B) were induced (with tetracycline) for 6 h to knock down TbNRP1, the cells were chilled on ice for 10 min, followed by addition of Tf-AlexaFluor594 for 15 min at 3 °C. Cells were then processed to detect (a) the presence of Tf-AF594, and (b) the distance between kinetoplast DNA (kDNA) and Tf-AF594 endosomes; endocytic vesicles populate a region between kDNA and the nucleus^48^.

**Figure 3:**
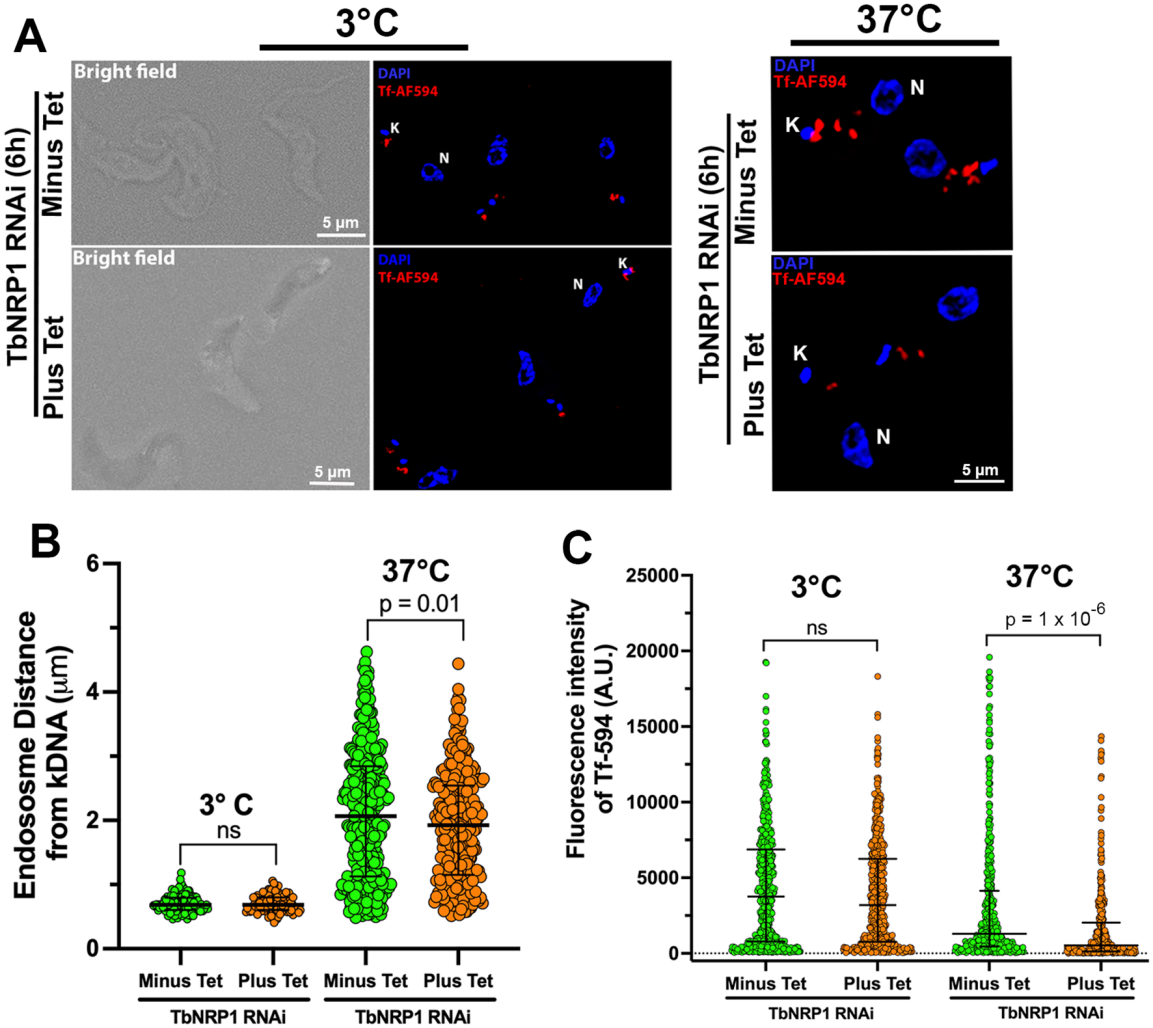
Binding (at 3 °C), and Uptake (at 37 °C) of Tf after knockdown of TbNRP1. **(A)** *Left panel*: TbNRP1 knockdown does not affect binding of Tf at flagellar pocket. Knockdown of NRP1 was induced for 6 h with 1 µg/mL tetracycline. Trypanosomes were incubated with Tf-Alexa594 (25 µg/ml) at 3 °C for 15 min. Cells were fixed with PFA, washed and mounted with Vectashield containing DAPI. Representative images of cells showing Tf-AF594 at the flagellar pocket region in uninduced (Minus Tet) and induced (Plus Tet) TbNRP1 RNAi cells. *Right panel:* Internalization of Transferrin is reduced after knockdown of TbNRP1. Knockdown of NRP1 was induced for 6 h with 1 µg/mL tetracycline. Trypanosomes were incubated with Tf-Alexa594 (25 µg/ml) at 37 °C for 2 min. Cells were fixed with PFA, washed and mounted with Vectashield containing DAPI. Images of cells showing internalized Tf-AF594 in uninduced (Minus Tet) and induced (Plus Tet) TbNRP1 RNAi cells. K, Kinetoplast; N, Nucleus. (**B**) Quantitation of motility of bound or internalized Tf. Scatter dot plot presents distance between kDNA, near flagellar pocket (site of endocytosis) and Tf at 3 °C or 37 °C in uninduced (Minus Tet) and induced (Plus Tet) cells. (**C**) Endosomes contain less Tf after knockdown of NRP1. Scatter dot plot represents Tf-AF594 fluorescence intensity (arbitrary units) in uninduced (Minus Tet) and induced (Plus Tet) cells at 3 °C or 37 °C. Bars denote median with interquartile range. *p* value was calculated using Kolmogorov–Smirnov test.

Tf-AF594 was observed at the flagellar pocket region in both uninduced and induced trypanosomes (Fig. 3A, Left panel). Measured from the kinetoplast, the distribution of Tf endosomes in both populations was centered around a median distance of ~ 0.7 µm (*p* = 0.96, Kolmogorov–Smirnov test, n = 3, 100 cells) (Fig. 3B). The fraction of trypanosomes with Tf at the flagellar pocket was 96.3% (uninduced), and 97.0%, (induced) (n = 3, 300 cells). The difference in the proportion of cells with Tf was not significant statistically (Supp. Fig. S2. We quantitated the amount of Tf at the flagellar pocket before and after knockdown of TbNRP1. In uninduced trypanosomes the median fluoresence intensity was 3757 (A.U.) compared to 3192 (A.U.) in induced trypanosomes. The difference in the distribution of Tf-AF594 fluoresence intensities between control and knockdown cells was not statistically significant (*p* = 0.12, Kolmogorov–Smirnov test) (Fig. 3C). We conclude that TbNRP1 is not important for binding of Tf to the flagellar pocket of *T. brucei*.

### Endocytosed Tf is reduced after knockdown of TbNRP1

Since impaired binding of Tf at the flagellar pocket (Fig. 3) could not explain reduced Tf endocytosis after knockdown of TbNRP1 (Fig. 2), we hypothesize that (1) vesicle formation, and/or (2) capture of Tf into endosomes was inhibited after knockdown of TbNRP1. To test these theories, TbNRP1 knockdown (or control) trypanosomes were induced with tetracycline for 6 and then incubated for 2 min with Tf-AF594 at 37 °C. Thereafter, trypanosome populations were analysed for (a) distribution of endosome distances from kDNA, and (b) intensity of Tf fluoresence in individual endosomes.

Following a 37 °C incubation, Tf-AF594 was detected in endosomes between kDNA and the nucleus (Fig. 3A, Right panel). Ordinarily, endosomes move from the flagellar pocket (near kDNA) towards the lysosome which is positioned close to the nucleus. As a result motility of endosomes can be quantitated by measuring distances between endosomes and kDNA. Median distances between Tf-containing endosomes and kDNA were 2.06 µm (control cells) and 1.92 µm (after knockdown of TbNRP1) (Fig. 3B). The difference in distribution of distances between kinteoplasts and Tf-positive endosomes before and after TbNRP1 knockdown was statistically significant (*p* = 0.01, Kolmogorov–Smirnov test, n = 3, 100 cells) (Fig. 3B). Thus, TbNRP1 knockdown reduces distances travelled away from the flagellar pocket by Tf endosomes. Lowering the temperature reduced endosome distances from kDNA. At 3 °C the median distance of endosomes was 0.686 µm (control cells), which increased threefold to 2.06 µm at 37 °C (Fig. 3B). The difference in distribution of Tf endosome distances between 3 and 37 °C was statistically significant (*p* < 1 × 10^−15^, Kolmogorov–Smirnov test, n = 3, 100 cells).

Endosomal Tf was quantitated by determining fluorescence intensity of individual vesicles before and after knockdown of TbNRP1. Following knockdown of TbNRP1 the median fluorescence decreased from 1,308 to 522 (A.U.) (Fig. 3C): the difference in distribution of fluoresence intensities between the two populations was statistically significant (*p* = 1 × 10^−6^, Kolmogorov–Smirnov test, n = 3, 300 cells). These data are consistent with TbNRP1 playing an important role in capture or/and retention of Tf in endosomes. Our data does not allow us to distinguish between TbNRP1 stimulation of robust Tf capture into endosomes from stabilization of Tf retention in vesicles.

### Endocytosed Tf colocalizes with TbNRP1 and clathrin

**Figure 4:**
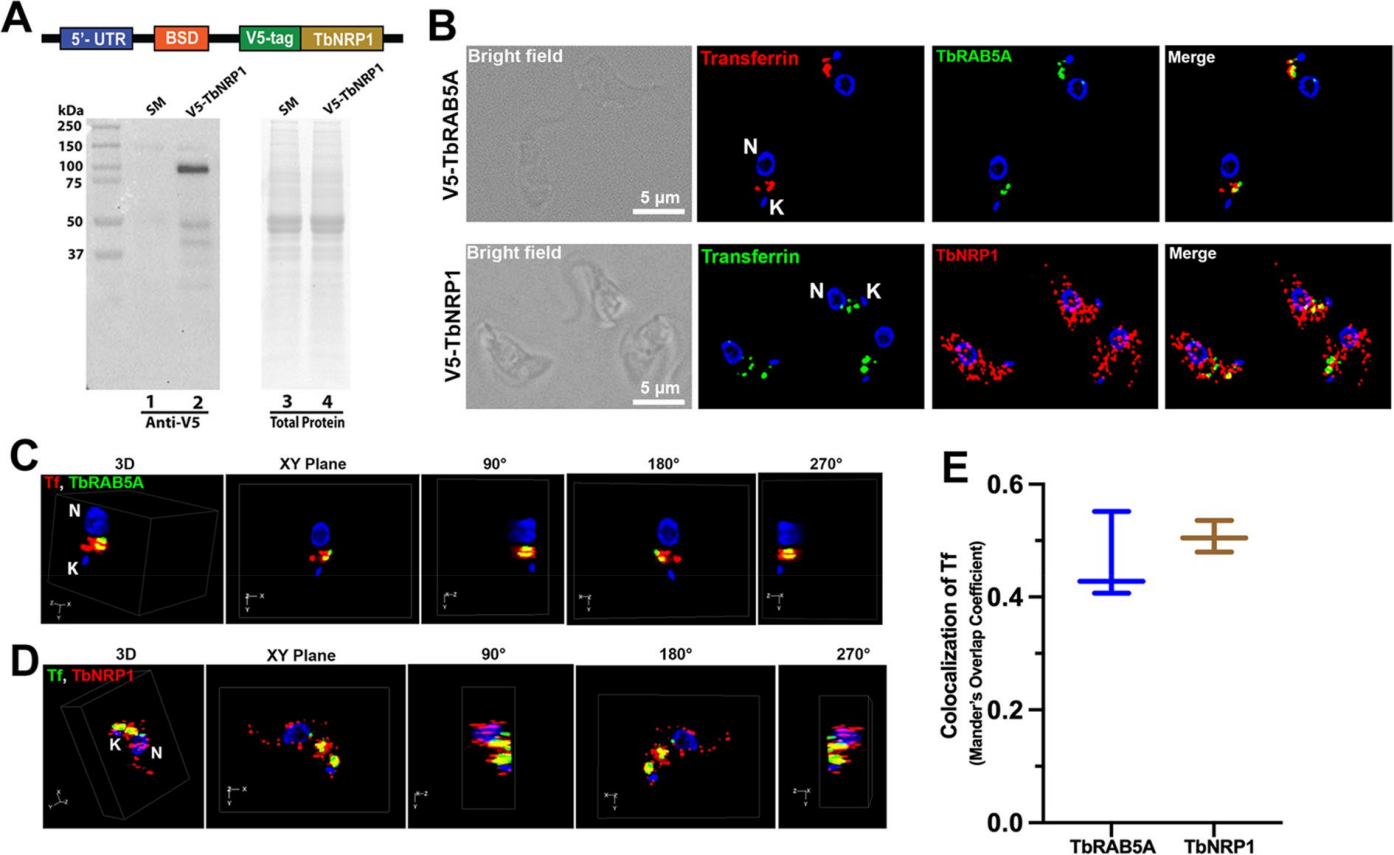
Endocytosed Tf co-localizes with V5-TbNRP1 (**A**) Western blot analysis of a V5-TbNRP1 trypanosome line. Trypanosomes (2 × 10^6^) were lysed, and proteins were separated by SDS-PAGE (12%). Proteins were transferred to a PVDF membrane that was developed using anti-V5 (rabbit) primary antibody. (**B**) Detection of Tf and intracellular V5-RAB5A or V5-NRP1. Cells were incubated for 2 min with Tf-Alexa594 (25 µg/mL) or Tf-Alexa488 (25 µg/mL) at 37 °C, fixed in PFA and stained with anti-V5 (rabbit) antibody. DAPI was used to detect DNA. Upper panel of images depict location of internalized Tf-AF594 and TbRAB5A. Lower panel of images show internalized Tf-AF488 and TbNRP1. (**C**) and (**D**) represent 3D reconstruction of Z-stack images of Tf and TbRAB5A or Tf and TbNRP1 respectively. (**E**) Graph shows overlap (Mander’s coefficient) of endocytosed Tf with TbRAB5A or TbNRP1. Three hundred cells were analysed from three independent biological samples. K, Kinetoplast; N, Nucleus.

Several hypotheses could explain how TbNRP1 facilitates capture/retention of Tf in endosomes (Fig. 3C). Since TbNRP1 lacks catalytic activity, we postulated that the protein exerts an effect on Tf endosomes by association with intracellular vesicles. We tested the hypothesis by investigating the localization of TbNRP1 inside *T. brucei*. For this purpose, an N-terminus V5 epitope-tagged TbNRP1 construct (V5-TbNRP1) (Fig. 4A) was transfected stably into an RNAi line (p2T7-TbNRP1). Immunoblotting confirmed the presence of V5-tagged TbNRP1 with the expected molecular weight (78 kDa) (Fig. 4A, lane 2). An immunofluoresence assay (IFA) showed V5-TbNRP1 association with cytoplasmic puncta (Fig. 4B). A portion of TbNRP1 was detected at the flagellar pocket region near BILBO1^49^ (Supp. Fig. S3), in methanol-fixed cells where most cytosolic proteins are extracted leaving behind cytoskeletal structures predominantly. This data is consistent with cytoskeletal association of a fraction of TbNRP1.

We examined possible association of TbNRP1 with Tf vesicles. RAB5A is an early endosome marker whose location overlaps Tf vesicles in *T. brucei*^50^. We used a V5 epitope-tagged Tb-RAB5A as control protein. An IFA of V5-TbNRP1 and endocytosed Tf (Fig. 4B) showed that a fraction of V5-TbNRP1 overlapped with Tf (Fig. 4B), and three-dimensional reconstruction of fluorescence images established co-localization (i.e., three-dimensional overlap) of Tf and NRP1 (Fig. 4D) ( see Video 1). The extent of co-localization between Tf and TbNRP1 was 0.5 (Mander’s overlapping coefficient^51^) (Fig. 4E); 50% of Tf co-localized with NRP1. Similarly, endocytosed Tf overlapped TbRAB5A (Fig. 4B,C) with a Mander’s coefficient of 0.42 (n = 300 cells) (4E). Conversely, the proportion of TbNRP1 overlapping with Tf (M2) was 0.04 (n = 300 cells) reflecting comparative excess of the pseudokinase in the cell.

**Figure 5:**
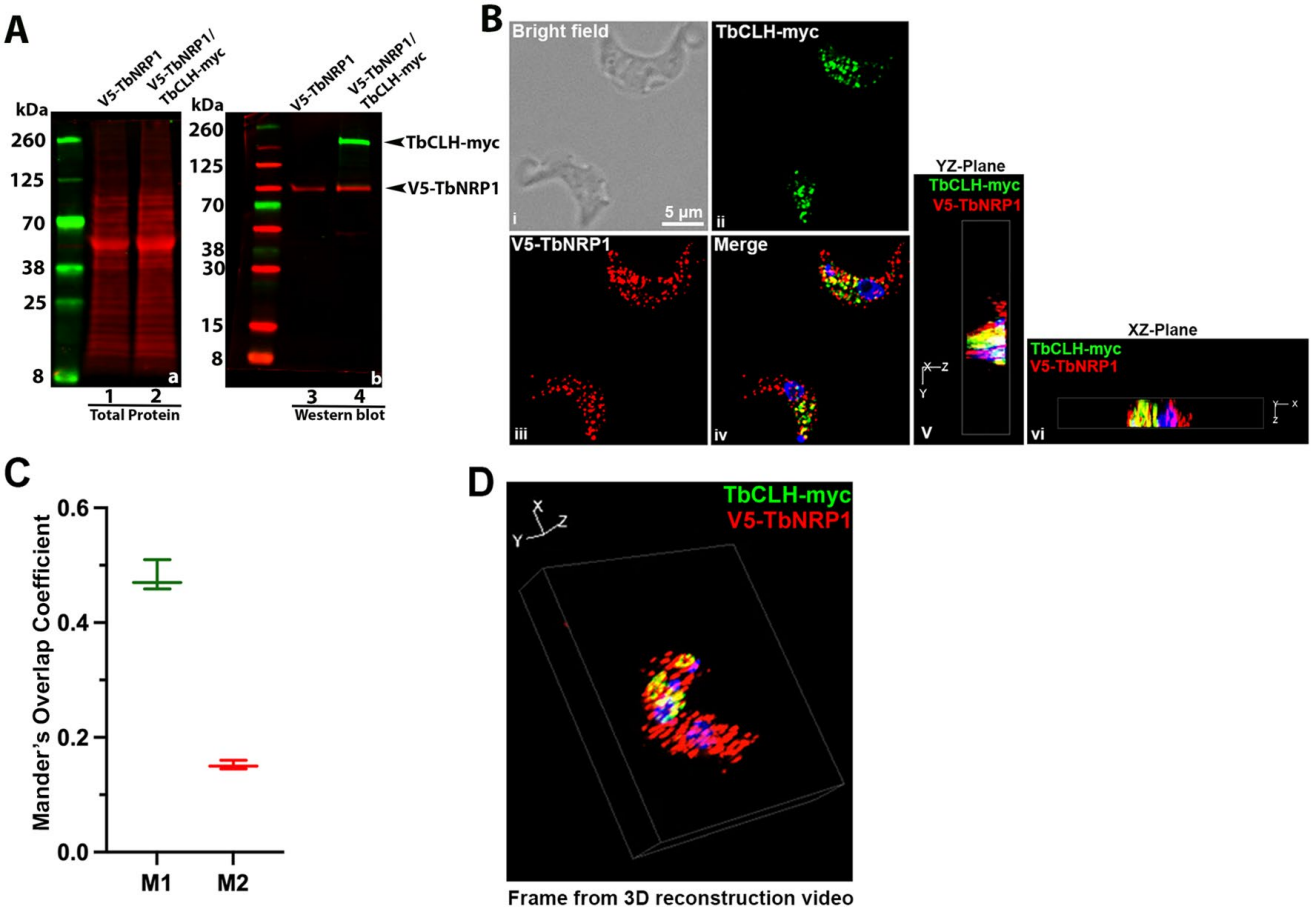
Clathrin Colocalizes with TbNRP1 (**A**) Western blot analysis of myc tag in V5-TbNRP1/TbCLH-myc cell line. Trypanosomes (2 × 10^6^) were lysed, and proteins were separated by SDS-PAGE (12%). Polypeptides were transferred to a PVDF membrane. The membrane was probed with anti-V5 (rabbit) or anti-myc (mouse) primary antibody and developed using goat anti-rabbit IR Dye 680 or goat anti-mouse IR Dye 800 (LI-COR) secondary antibody for near-infrared based fluorescence detection. (**B**) Detection of CLH-myc and NRP1. Cells were fixed in PFA and stained using anti-V5 (rabbit) and anti-myc (mouse) antibodies. DAPI was used to stain DNA. (i) (ii) and (iii) represent bright field, CLH-myc and V5-NRP1 locations respectively, and (iv) is a merged fluorescence image of TbCLH-myc and V5-TbNRP1. Panels (v) and (vi) are merged images in different planes (XY, XZ). (**C**) Box and Whisker plot of overlap analysis (Mander’s coefficient) between CLH-myc and V5-NRP-1. M1 is the extent of overlap between CLH-myc and NRP1, whereas M2 is degree of overlap between NRP1 and CLH-myc. A total 300 cells from three independent biological experiments were analysed. (**D**) A frame from 3D reconstruction video (see Video 2). K, Kinetoplast; N, Nucleus.

Clathrin is a major vesicle coat protein for endocytosis in eukaryotes^52,53^. Since clathrin is involed in endocytosis in *T. brucei*^54,55^ we examined the possiblility of co-localization of clathrin and TbNRP1. Here V5-tagged TbNRP1 cell lines were transfected with myc-tagged clathrin heavy chain (CLH) to generate a V5-NRP1/CLH-myc trypanosome line (Fig. 5A). Trypanosomes were then processed to detect intracellular NRP1 and clathrin using double-immunofluorescence microscopy (Fig. 5B). From Mander’s overlap coefficient analysis, fifty percent of CLH co-localized with NRP1 (M1 = 0.5) (Fig. 5C), and seventeen percent of NRP1 co-localized with CLH (0.17 (M2) (n = 300 cells)) (Fig. 5C). 3D IFA results confirmed these observations (Fig. 5D) (see Video 2). We conclude that clathrin co-localizes with NRP1.

### Knockdown of TbNRP1 alters the phospho-proteome of T. brucei

Pseudokinases can modulate activity of protein kinases^56–60^, among other modes of influencing biological pathways^61,62^. In addition, endocytosis in eukaryotes is controlled by phosphorylation of proteins^19,26,63–65^. Here, we tested a hypothesis that knockdown of TbNRP1 perturbs the phospho-proteome of *T. brucei*.

To discover changes in the trypanosome phospho-proteome, stable isotope labelling of amino acids in cell culture (SILAC)^66^ was performed^67^, followed by enrichment of phosphopeptides using immobilised metal affinity chromatography (IMAC)^68^, and identification of peptides by mass spectrometry^31,69^. Trypanosomes (p2T7-TbNRP1) were cultured in heavy (^13^C_6_-L-Arginine, ^2^H_4_-L-Lysine) or light isotopes (L-Arginine, L-Lysine) for SILAC. Trypanosomes grown in heavy medium were induced with tetracycline to knock down TbNRP1. Induced (Tet + , H) and uninduced (Tet-, L) samples were combined and processed together for IMAC and mass spectrometry. Phospho-peptides whose abundance changed two-fold (at least) in two of more independent biological replicates were presented if their posterior error probability (PEP-value)^70^ was below 10^−3^ (Table 1). These phospho-peptides identify proteins affected after TbNRP1 knockdown and we termed them “NRP1-pathway proteins” (Table 1).

**Table 1:** Select TbNRP1-pathway proteins.

Gene ID	Description	Phosphopeptide Sequence	Fold change (± std dev), n ≥ 2	Qvality PEP value
**Decrease**				
Tb247.06.4390	Kinesin heavy chain isoform 5B (KHC1)	DGTPsPNNTQNENLQR	2.9 ± 0.06	1.9e^−05^
Tb427.10.5880	Proteophosphoglycan	AsVSEEANNVSSDRPVGK	2.7 ± 0.07	5.0e^−05^
Tb427.08.8000	Hypothetical	DsLFADGGELDsFYAK	2.6 ± 0.12	1.2e^−06^
Tb427.10.15040	Hypothetical	SGtCVVNLAESTK	2.5 ± 0.06	4.2e^−03^
Tb427tmp.01.1960	Hypothetical	KTsSAPsLLPQIK	2.4 ± 0.02	1.1e^−03^
Tb427.04.2220	TPR-repeat-containing chaperone protein DNAJ	LSILGDITAEPLsAR	100 ± 0	1.4e^−05^
Tb427.07.3740	Hypothetical	GMsPEDsNNPESLFVR	100 ± 0	1.1e^−05^
Tb427.08.1300	Hypothetical	SAAACANtsMETTPEAVNR	100 ± 0	2.5e^−04^
Tb427tmp.160.4710	Hypothetical	sCEEVGSPDQAVNDSYVQLER	100 ± 0	4.9e^−09^
Tb427tmp.211.4170	Hypothetical	GAAAGDItPPQDEAEK	100 ± 0	1.5e^−08^
Tb427.05.1690	Hypothetical	ELQQQLSStAVAR	100 ± 0	1.2e^−04^
**Increase**				
Tb427.07.5450	Lipin	GLEMSGMsNPSASVAVTNR	2.41 ± 0	1.8e^−05^
Tb427tmp.50.0006	EpsinR	AGITVsEAQR	2.34 ± 0.01	1.5e^−03^
Tb427.07.3000	Kinesin	LSVADSSPSTHSPSPTEsPTVR	2.99 ± 0.25	6.4e^−05^
Tb427.06.640	Phospho-protein phosphatase	AAPSANVsSVTSPPR	51.0 ± 68.84	7.24e^−07^
Tb427.08.3770	MAP Kinase	EDTQDPNKtHYVTHR	2.4 ± 0.03	6.5e^−03^
Tb427.05.2820	NEK6, protein kinase	MCsPANSPVSPSR	2.5 ± 0.34	2.2e^−04^
Tb427.05.3560	VAMP, Synaptobrevin	SAtLSEQAQQFQR	3.84 ± 0.83	1.8e^−04^
Tb427tmp.02.0260	Golgi reassembly stacking protein	VPPPLAFPIIKPAtPSR	2.03 ± 0.04	1.8e^−03^
Tb427.05.2410	Kinesin	IALsGATGDLMK	2.28 ± 0.25	1.7e^−05^
Tb427tmp.02.4140	IP3-5-phosphatase	FPPTYLCQsPR	51.15 ± 69.1	5.5e^−05^
Tb427.07.5220	Forkhead Kinase	GDICGDFsDAEDGDTSSAVR	3.0 ± 1.0	2.6e^−07^
Tb427.01.4310	Hypothetical	TGAtPLR	100 ± 0	5.8e^−03^
Tb427.02.4050	Hypothetical	GEQDIAVVSSREDDVK	100 ± 0	3.7e^−03^
Tb427.03.1920	NOT5 protein	GRPASLVsPPSTTSK	100 ± 0	8.8e^−04^
Tb427.04.3970	Hypothetical	KHELLLsPPEAEK	100 ± 0	1.0e^−04^
Tb427.06.840	Hypothetical	HESSSIMGNsPPDSK	100 ± 0	4.2e^−09^
Tb427.07.1110	Asparagine synthetase a	APDYDDWtSPVEASQVVFPR	100 ± 0	1.4e^−05^
Tb427.07.7240	Hypothetical	DCATPSAGGYAGSGPsGR	100 ± 0	5.7e^−07^
Tb427.07.7270	Hypothetical	FALGEVLAPsPLR	100 ± 0	2.6e^−04^
Tb427.10.14410	Hypothetical	DTAVAtPDAAEAADSQYNR	100 ± 0	1.3e^−06^
Tb427tmp.01.4480	Hypothetical	LSKPQQPSNSSGGDsK	100 ± 0	1.6e^−03^
Tb427tmp.160.0340	TbMlp-2, Myosin-like protein	EVEELGGSSGPsSAR	100 ± 0	4.8e^−07^
Tb427tmp.160.4210	Hypothetical	VsPINDSIPETGQEEQEIGEISPR	100 ± 0	1.4e^−05^
Tb427tmp.39.0006	eIF-2B GDP-GTP exchange factor	FSADDLFAsLR	100 ± 0	1.7e^−04^

Phospho-peptides whose abundance changed two-fold (at least) in two of more independent biological replicates in mass spectrometry experiment (see materials and methods) were presented, if their posterior error probability (Qvality PEP-value) was below 10^−3^. These phospho-peptides identify proteins affected after TbNRP1 knockdown.

**Figure 6:**
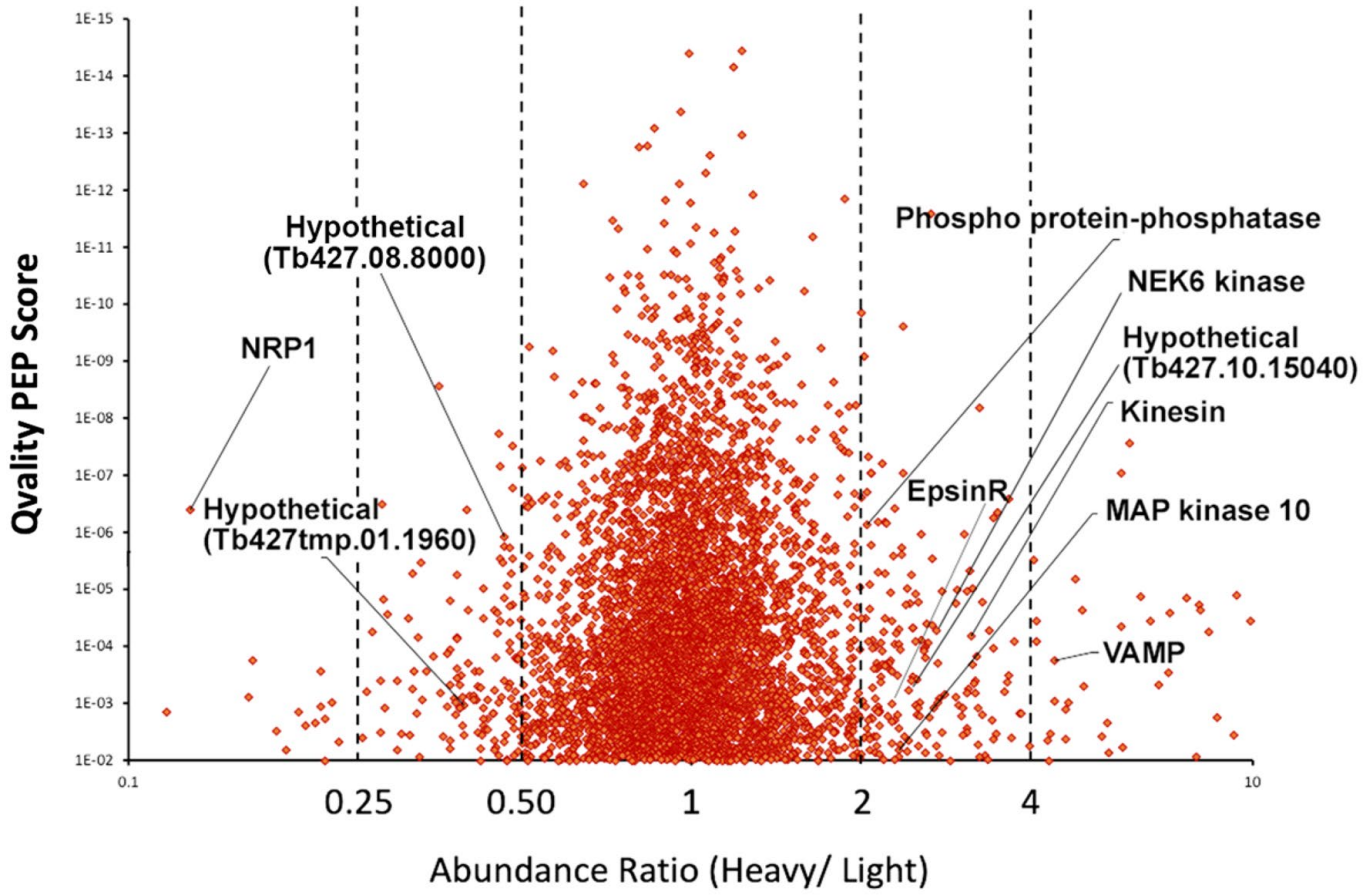
Knockdown of TbNRP1 alters phosphoproteome of *T. brucei*. TbNRP1-RNAi trypanosomes were labeled in heavy (H) or light (L) HMI-9 SILAC media prior to induction of knockdown with tetracycline 1 ug/mL (Tet+). Twenty-four hours after additional of tetracycline, 4 × 10^7^ cells were mixed, lysed, and trypsin digested. Peptides were adsorbed to a SepPak C18 column. Phosphopeptides were enriched by immobilized metal affinity chromatography (IMAC) followed by LC–MS/MS to identify the sequences. Representative proteome profiles of knockdown trypanosomes (Tet+) or uninduced cells (Tet−). The abundance ratio (H/L) of identified phosphopeptides is plotted as a function of their Qvality posterior error probability (PEP) score.

TbNRP1 knockdown affected 202 proteins; 62 decreased in phosphorylation, while 140 were hyper-phosphorylated (Fig. 6). Three protein kinases were affected by knockdown of TbNRP1, namely forkhead kinase (Tb427.07.5220), NEK6 (Tb427.05.2820) and mitogen activated protein kinase 10 (Tb427.08.3770) (Table 1). In addition, proteins that regulate Tf endocytosis (EpsinR (Tb427tmp.50.0006)), and others predicted to be involved is aspects of endocytosis were affected, namely kinesins (Tb427.06.4390, Tb427.07.3000, and Tb427.05.2410)^71^, and VAMP (Tb427.05.3560). This set of proteins offer an opportunity in future work to link noncatalytic roles of TbNRP1 to either protein kinases or to vesicle-associated proteins that regulate endocytosis.

## Discussion

### Discovery of new proteins for Tf endocytosis in a Trypanosome

Proliferation of bloodstream *T. brucei* in a vertebrate host requires iron^72,73^, obtained by receptor-mediated endocytosis of transferrin (Tf), a host protein^4^. Drugs that inhibit endocytosis of Tf also kill *T. brucei*^25,26,31,35^, suggesting that proteins required for uptake of Tf may be targets for design of new anti-trypanosome drugs.

Many aspects of the Tf endocytosis pathway are not understood. The receptor for Tf (TbTfR) is atypical compared to its vertebrate counterpart^7,74,75^. First, there is no significant sequence similarity between the two proteins. Second, TbTfR is attached to the plasma membrane by a GPI anchor^76–78^. Absence of cytoplasmic segments of GPI-anchored receptors has hampered identification of polypeptide factors used in their endocytosis (reviewed in^79–81^).

For a comprehensive molecular account of the Tf endocytosis pathway in a trypanosome, it is important to identify proteins, besides TfR and EpsinR that are used for endocytosis of that cargo. Efforts to use vertebrate protein sequence alignments to identify trypanosome counterparts of the Tf endocytosis pathway can only be partially successful^71^, due to extensive divergence of trypanosome and vertebrate protein sequences^82,83^. Given these constraints, an unbiased function-driven strategy may be a useful complement to bioinformatics. We used the pyrrolopyrimidine AEE788^34^, a kinase inhibitor, to perturb endocytosis of Tf, and then analyzed the phosphoproteome^35^. AEE788 caused hyper-phosphorylation of NRP1 under conditions where it inhibited Tf endocytosis, suggesting that the protein could be involved in endocytosis. In agreement with this hypothesis, knockdown of TbNRP1 inhibited Tf endocytosis (Fig. 2).

### Hypothesis: how a pseudokinase may regulate Tf endocytosis in *T. brucei*

Pseudokinases affect diverse cellular processes^84^. Pathways for kinase-independent control of biological and biochemical processes (reviewed in^61,85,86^) include allosteric activation (or inhibition) of related active protein kinases (e.g., pseudokinase JH2 attenuation of JH1 in the Janus Kinase (JAK) pathway)^87,88^. Pseudokinase HER3 modulates signaling by protein kinases HER2 and HER1^43^, whereas the pseudokinase Kinase Suppressor of Ras (KSR) heterodimerizes with Raf kinases to activate them^89,90^. In the necroptosis pathway, the Mixed Lineage Kinase domain-like pseudokinase (MLKL) oligomerizes to disrupt the plasma membrane after phosphorylation by RIP3 kinase^91–93^. Elsewhere SCYL2 influences clathrin vesicle transport^94^. Thus, the noncatalytic activity of a pseudokinase can modulate integrity of biological membranes.

Trypanosome NRP1 is a pseudokinase lacking both HRD and DFG motifs present in catalytic protein kinases (reviewed in^42^). NRP1 colocalizes with Tf endosomes (Fig. 4; Video 1) as well as the vesicle coat protein clathrin (Fig. 5; Video 2) and is important for stable internalization of Tf into vesicles (Figs. 2, 3C). How could NRP1 influence endocytosis of Tf? Biological effects of TbNRP1 may arise from (1) interaction with catalytic protein kinases (as illustrated above for JAK kinase)^87,88^ or (2) intrinsic activity of the protein (c.f. MLKL disruption of biomembranes, or SCYL2 effect on clathrin vesicles)^91–94^.

### Possible contributions of NRP1 pathway proteins to endocytosis

Knockdown of NRP1 (Fig. 6) resulted in dephosphorylation of 62 polypeptides and hyperphosphorylation of 140 were polypeptides. We term these 202 proteins “NRP1-Pathway Proteins” (Table 1). Compared to proteins perturbed after addition of AEE788 to trypanosomes, five proteins were common to both treatments (Table 2). We speculate that these five proteins impact endocytosis of Tf in *T. brucei*.

**Table 2:** Proteins affected by both treatment of trypanosomes with AEE788 and knockdown of TbNRP1.

Gene ID	Description	Phosphopeptide Sequence	Fold Change (± std dev), n ≥ 2	Qvality PEP value
**Decrease**				
Tb427tmp.160.4770	TbNRP1	DEAAASsVKsCTAAQ	14 ± 0.01	5.0e^−10^
Tb427.08.8000	Hypothetical	DsLFADGGELDsEYAK	2.6 ± 0.12	1.2e^−06^
Tb427.10.15040	Hypothetical	SGtCVVNLAESTK	2.5 ± 0.06	4.2e^−03^
Tb427tmp.01.1960	Hypothetical	KTsSAPsLLPQIK	2.4 ± 0.02	1.1e^−03^
**Increase**				
Tb427.05.2820	NEK6, protein kinase	MCsPANSPVSPSR	2.5 ± 0.34	2.2e^−04^

Proteins shown in the table are TbNRP1-pathway proteins that were also affected by treatment of trypanosomes with AEE788 for 9 h. AEE788 data used to prepare the table was obtained from Sullenberger et al., 2017.

Protein kinases whose phosphorylation was perturbed after NRP1 knockdown included Nek6, Forkhead, and MAPK10 (Table 1). Forkhead kinase and DNAJ both associate with endosome-like foci, from data available from TrypTag^95,96^, raising a possibility, because of their intracellular location, that those proteins impact uptake of Tf.

Since endocytosis depends on intracellular vesicles, we searched for vesicle-associated proteins among NRP1-pathway proteins and we found tubulin^97,98^, EpsinR^71,99^, Synaptobrevin^100,101^, myosin-like 2^102,103^, GRASP^104–106^, and kinesins^107,108^ (Table 1). Data available at TryTag^95,96^ indicates that synaptobrevin associates with endosome-like structures found between the nucleus and kinetiplast. EpsinR regulates endocytosis of Tf^71^, and could be a link between NRP1 and the Tf uptake system: New experiments are needed to directly address hypotheses postulated in this section.

## Methods and materials

### Cell culture

Bloodstream *Trypanosoma brucei brucei* Single Marker (SM)^44^ line was maintained at logarithmic phase (< 10^6^/mL) in HMI-9 medium^109^ containing G418 (6.5 µg/mL). SM trypanosomes produce T7 RNA polymerase and tetracycline repressor for conditional induction of genes under T7 promoter control after tetracycline induction. An RNAi line for TbNRP1 knockdown was maintained in HMI-9 with G418 (6.5 µg/mL) and hygromycin (5 µg/mL)^44^. A V5-tagged TbNRP1 line was maintained in HMI-9 containing G418 (6.5 µg/mL), hygromycin (5 µg/mL), and blasticidin (5 µg/mL)^110^.

### Generation of p2T7-TbNRP1 RNAi vector

Unique fragments of 527 bp and 566 bp length against different regions of TbNRP1 were obtained using RNAit^111^. Primers for amplification of the double-stranded DNA fragment were: FP-1: 5′-GGATCCTTCTGCTTCTCGCAGACTGAGCGG-3′, RP-1: 5′-CTCGAGAAGAGGTCATCCGTTGTTGGTTTTTGAGGCT-3′, FP-2: GGATCCTCGCGATAAGGGAATCCTGC-3′, RP-2: 5′-CTCGAGCCGTGTACCACACTTCAGCT-3’. Purified genomic DNA (100 ng)^112^ was used as template for PCR to facilitate cloning into RNAi vectors. A 5’ *BamH1* site, 5′-GGATCC-3′ and a 3′ *Xho1* site, 5′-CTCGAG-3′ were included in the primers (underlined in respective primer sequences, above). Following PCR product “clean-up” (Qiagen) and addition of 3′-A overhangs (Invitrogen pCR™8/GW/TOPO® TA Cloning® Kit), the amplified fragment was cloned into pCR®8/GW/TOPO and the resulting plasmid was digested using *BamH1* and *Xho1* (New England Biolabs). A released TbNRP1 fragment, and a p2T7^TABlue^ RNAi vector^45^ were digested, separated, and extracted from agarose gel (Qiagen). Following phosphatase treatment of the vector it was ligated to PCR product using T4 DNA ligase (New England Biolabs) to produce a p2T7-TbNRP1 RNAi construct.

### Endogenous N-terminus V5 tagging of Tb427tmp.160.4770 (TbNRP1)

V5-epitope tagging of TbNRP1 was carried out by homologous recombination^110^. PCR primers used were: Forward: 5′-TACTTAGGTCAGTACCATAGTTTGGCGAGGTCACTGAAGTATTCCTCACACGAAGAAAGGAAGAAACAGAGAAGAAAAGTGAAGGAATAACCCGGGATGGCCAAGCCTTTGTCTCAAGAAG -3′, and Reverse: 5′-GGTACCATCCTTGTTATACTTCTCAATTTTACCCTTTGATGAGAAGTTCTTGTCCTTGGACTTACTAGATTTACTATCACTGCCCCCGGGCGTAGAATCGAGACCGAGGAGAGGGTTAG -3′. DNA amplification was carried out with iProof high-fidelity PCR polymerase master-mix (Bio-rad, Hercules, Ca), yielding a product with the 5′ UTR of TbNRP1, followed by a blasticidin resistance gene, then a fusion of the V5-epitope to the beginning of the 5’ coding sequence of TbNRP1. The PCR product was nucleofected into a p2T7-TbNRP1 RNAi line, and drug-resistant clones were selected as described^113^.

### Trypanosome transfection

Single-marker trypanosomes were cultured in HMI-9 medium to a density of 8 × 10^5^ cells/mL. For transfection, 4 × 10^7^ cells were pelleted (3000 x*g* for five minutes), washed in 10 mL of 10 mM glucose in phosphate buffered saline (PBSG), and resuspended in 100 µL of Lonza Nucleofector solution (Lonza, Basel, Switzerland) with 20 µg of NotI-linearized p2T7-TbNRP1. Nucleofection was performed in 2 mm cuvettes in a Lonza Nucleofector device using program code “T cell (CD4 +) mouse”. Eighteen hours after nucleofection, trypanosomes were serially diluted (1:10, 1:100, 1:1000) in HMI-9 medium, seeded in 24-well plates, and selective drugs added^26^.

### Cell proliferation assays

Trypanosomes (p2T7-TbNRP1) were inoculated in a 24-well plate at 1 × 10^4^ cells/mL, and induced with tetracycline (1 µg/mL) for RNAi. Cell density was determined with a haemocytometer every 12 h over a 36-h period. Three independent biological experiments each with technical replicates were performed.

### Endocytosis assays

Effects of TbNRP1 knockdown on endocytosis of protein cargo, fluorescent transferrin-AlexaFluor488 (Tf-Alexa488, 25 mg), bovine serum albumin-AlexaFluor647 (BSA-Alexa647, 25 mg) or haptoglobin-hemoglobin-AlexaFluor647 (Hp-Hb-Alexa647, 30 mg), was determined with flow cytometry^26,35^ [(12,000–15,000 events per sample) in three biological samples with technical duplicates. Standard deviation and statistical significance of differences in mean measurement between treatment groups and the control was determined with a t-test (GraphPad Prism (version 7.0c)].

### Western blot analysis

Trypanosomes (10^6^) were pelleted (3000 x*g*, five minutes), washed in PBSG, resuspended in 15 µL of SDS sample buffer, and heated at 95 °C for five minutes. Proteins were separated by SDS-PAGE (12% acrylamide) and labelled with Stain-Free probe (Bio-Rad, Hercules, Ca). The stain-free gel was UV-activated for five minutes before transfer (semi-dry rapid transfer; Bio-Rad, Hercules, CA) to a PVDF membrane that was was blocked in 5% milk in TBS-T (20 mM Tris buffered saline with 0.1% Tween-20) for 1 h. Membranes were probed with primary antibodies rabbit anti-V5 (Cell Signalling Technology; 1:2000) in 5% bovine serum albumin (BSA) in TBS-T for 1 h. PVDF membranes were washed three times, five minutes each with TBS-T at room temperature, and then incubated for one hour with alkaline phosphatase conjugated goat anti-rabbit IgG secondary antibody (Bio-Rad, Hercules, CA) diluted 1:2000 in 5% BSA in TBS-T. Excess secondary antibody was washed off, and the membrane developed for three minutes with Immune-Star AP substrate (Bio-Rad, Hercules, CA). Digital images of membranes were captured with a ChemiDoc MP system (Bio-Rad). Normalization of western blots was performed against the total protein loaded per lane using Image Lab software (Bio-Rad).

For near-infrared based western blot detection of V5-TbNRP1 and TbCLH-myc , membranes were probed with primary antibodies rabbit anti-V5 (Cell Signalling Technology; 1:2000); mouse anti myc monoclonal 9E-10 (Santa Cruz Biotechnology; 1:1000) in 5% bovine serum albumin (BSA) in TBS-T for 1 h. PVDF membranes were washed three times, five minutes each with TBS-T at room temperature, and then incubated for one hour with goat anti-rabbit IR Dye 680 and goat anti-mouse IR Dye 800 (LI-COR) secondary antibody (1:20,000 dilution in 5% milk in PBS). Membranes were scanned for fluorescence digital images on ODYSSEY CLx (LI-COR) and data was processed in Image Studio Software (LI-COR) .

### Fluorescence microscopy

Fluoresence of each endosome was monitored and quantitated as described in this section. To track coincidence of endocytosed Tf and TbRAB5A or Tf and TbNRP1, trypanosomes (10^6^) were pelleted, washed in 1 mL of serum-free medium, and incubated at 37 °C for 15 min in serum-free medium. Fluorescent transferrin (Tf-AlexaFluor-594 or Tf-AlexaFluor-488, 25 µg/ml, Life Technologies) was added to trypanosomes that were incubated for 2 min at 37 °C in a CO_2_ incubator. Cells were pelleted at 4 °C (3000 x*g*, five mins) and fixed in 4% paraformaldehyde (PFA) (Electron Microscopic Sciences) in PBS for 20 min. Fixed trypanosomes were adhered to poly-L-lysine coated coverslips and the fixative was quenched with 0.1 M glycine in PBS for ten minutes at room temperature. Trypanosomes were permeabilized with 0.5% Triton X-100 in PBS for five minutes at room temperature. Coverslips were washed briefly in PBS before blocking with 1% BSA (Sigma Aldrich), 1 X fish gelatin (Biotium Inc.) in PBS (blocking buffer) for one hour at room temperature. Primary antibody (rabbit anti-V5, Cell Signalling Technologies, 1:1000) adsorption was performed in blocking buffer for one hour. Coverslips were washed thrice in PBS (five minutes each time) followed by incubation with secondary antibody (goat anti-rabbit AlexaFluor-594 or goat anti-rabbit AlexaFluor-488 (Invitrogen), 1:1000) in blocking buffer for one hour at room temperature. Coverslips were washed, as described above, and mounted on glass slides with antifade mounting medium containing DAPI (Vectashield, Vector Laboratories).

Images were acquired using a BZ-X810 fluorescence microscope (Keyence Inc.) using a 60X objective, NA1.4. Images and videos were analysed and processed using BZ-X800 analyser software (Keyence Inc.). Total number of cells used for colocalization analysis was ~ 300 in three sets of independent experiments. Colocalization analysis was performed using Fiji^114^ and a colocalization tool plugin JACoP^115^.

For V5-TbNRP-1 and TbCLH-myc localization, anti-myc (9E10, Santa cruz biotechnology, 1:1000,) and anti-V5 (rabbit, Cell Signalling Technologies, 1:1000,) primary antibodies were used. Image acquisition, and coincidence analysis was performed as described above. In the V5-TbNRP1 and mNG-TbBILBO1 experiment, samples were prepared as described above, except that the cells were fixed and permeabilised with methanol (pre chilled in -20 °C) (Fisher Chemical) for 5 min.

For flagellar pocket binding of fluorescent transferrin, trypanosomes (10^6^) were pelleted, washed and incubated at 37 °C for 15 min in serum-free media. Cells were chilled on ice for 10 min followed by addition of Tf-Alexa594 (25 µg/ml) and incubation for 15 min at 3 °C. Cells were washed twice with ice-cold PBS at 4 °C, fixed with 4% PFA, washed with PBS and mounted on glass slides as described above. Images were acquired on a BZ-X810 fluorescence microscope (Keyence Inc) using a 60X objective, NA1.4. Total number of cells used for the measurement of fluoresence intensity was ~ 300 in three sets of biological experiments. To quantitate fluorescence intensity of Tf-594, a hybrid cell count module of BZ-X800 analyzer (Keyence) was used. Fluorescence intensity value (A.U.) shown in the graph represents sum of the pixel values in the extracted area of the image (target area intergration value). The distance between Tf endosomes and kinetoplast DNA (kDNA) was determined using a line tool in Fiji^114^. Stastistical analyses (Kolmogorov–Smirnov test) were performed with GraphPad Prism (version 7.0c).

### SILAC phospho-proteomics

Trypanosomes (p2T7-TbNRP1) were cultured in heavy (H) medium (^13^C_6_-L-Arginine, ^2^H_4_-L-Lysine), and control (uninduced) trypanosomes were grown in light (L) medium (L-Arginine, L-Lysine) for five days^116^. Trypanosomes grown in heavy medium were induced with tetracycline for twenty-four hours (see earlier description). Trypanosomes (4 × 10^7^/sample) were pelleted (3000 x*g*, five mins) and washed in 10 mL of PBSG containing 1 mM sodium orthovanadate (Na_3_VO_4_). The Tet + (H) sample was then combined with control Tet- (L) sample (4 × 10^7^ cells/sample). The cells were lysed and peptides obtained for mass spectrometry as described^31^.

## Supplementary Information

Supplementary Information 1.

Supplementary Video 1.

Supplementary Video 2.

## Data Availability

The mass spectrometry proteomics data have been deposited to the ProteomeXchange Consortium via the PRIDE [1] partner repository with the dataset identifier PXD034516.
