# Supplementary material for: Pseudokinase NRP1 facilitates endocytosis of transferrin in the African trypanosome
Source: Sci Rep. 2022 Nov 3;12:18572. doi: 10.1038/s41598-022-22054-x (PMC9633767; doi:10.1038/s41598-022-22054-x)

**Pseudokinase NRP1 Facilitates Endocytosis of Transferrin in the African Trypanosome**

Gaurav Kumar^1^, Bryanna Thomas^2^, and Kojo Mensa-Wilmot*^1,2^

^1^Department of Molecular and Cellular Biology, Kennesaw State University, Kennesaw, Georgia

^2^Center for Tropical and Emerging Global Diseases, University of Georgia, Athens, Georgia

*Corresponding author, E-mail: [kmensawi@kennesaw.edu](mailto:kmensawi@kennesaw.edu)

**Supplementary Figure 1.** TbNRP1 knockdown does not affect distribution of K/N in *T. brucei*. Knockdown of TbNRP1 was induced for 6 hours with 1 µg/mL tetracycline. Cells were fixed with 4% PFA, washed and mounted with DAPI containing Vectashield. Graph presents percentage of cells containing number of kinetoplasts (K) and nuclei (N) per trypanosome in absence or presence of tetracycline (1 µg/mL) for 6 h. Error bars represent standard deviation of three biological samples. Statistical analysis was performed using Chi-squared (x^2^) test.

**Supplementary Figure 2**. TbNRP1 knockdown does not affect flagellar pocket binding of Tf at 3°C. Knockdown of TbNRP1 was induced for 6 hours with 1 µg/mL tetracycline. Cells were incubated with Tf-Alexa594 (25 µg/ml) at 3^0^C for 15 minutes. Cells were fixed with 4% PFA, washed and mounted with DAPI containing Vectashield. Graph presents percentage of Tf-AF594 bound cells from minus Tet and plus Tet samples. Statistical analysis (α = 0.05) was performed using Unpaired Student’s t-test.

**Supplementary Figure 3**. Detection of TbNRP1 near flagellar pocket region in methanol-fixed cells. Trypanosomes were fixed and permeabilized in cold methanol, stained for V5 tag and mounted with vectashield containing DAPI. Endogenously tagged fluorescent mNeonGreen mNG-TbBILBO1 was used as a marker for the flagellar pocket. (i) Bright field image of trypanosomes, (ii) and (iii) shows localization of mNG-BILBO1 and V5-NRP-1 respectively. Panel (iv) shows merged panel ii and iii. K, Kinetoplast; N, Nucleus.

**Supplementary Figure 4**. Full-length Stain-Free gel and western blot corresponding to cropped images shown in Figure 1C. Samples were loaded in two sets: the dotted rectangle shows the cropped area presented in Figure 1C.

**Supplementary Figure 5.** Full-length Stain-Free gel and western blot from which the cropped images shown in Figure 4A was obtained. Protein samples were loaded in two sets, and the dotted line outlies the cropped area used to produce Figure 4A.

**Supplementary Figure 6.** Full-length gel and western blot corresponding to the cropped image shown in Figure 5A. Protein samples were loaded in two sets for electrophoresis, and the dotted rectangle highlights the area cropped for use in Figure 5A.

**Supplementary Figure 7.** This presents Supplementary Figure 6 in gray scale to show more clearly the edges of gel and membrane.

**Video 1**. A 3D reconstruction movie showing the presence of Tf-AF488 and V5-TbNRP1. Movie was created using 3D display module of BZ-X800 analyser software (Keyence).

**Video 2**. A 3D reconstruction movie showing the presence of Tf-AF594 and V5-TbRAB5A. Movie was created using 3D display module of BZ-X800 analyser software (Keyence).

**Supplementary Figure S1**

**
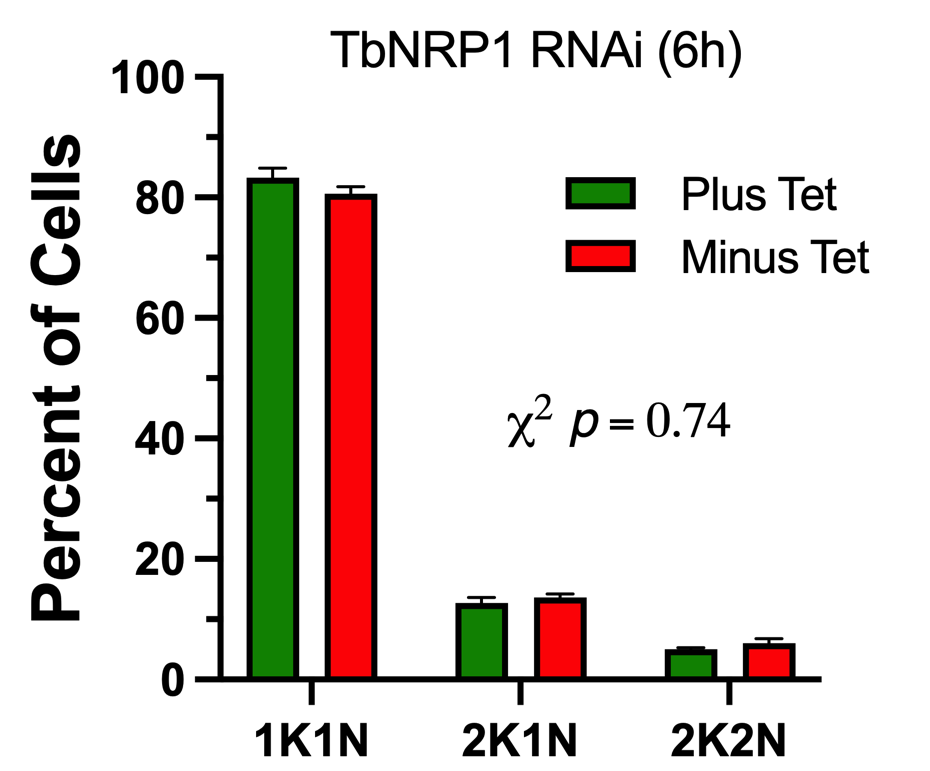
**

**Supplementary Figure S2.**


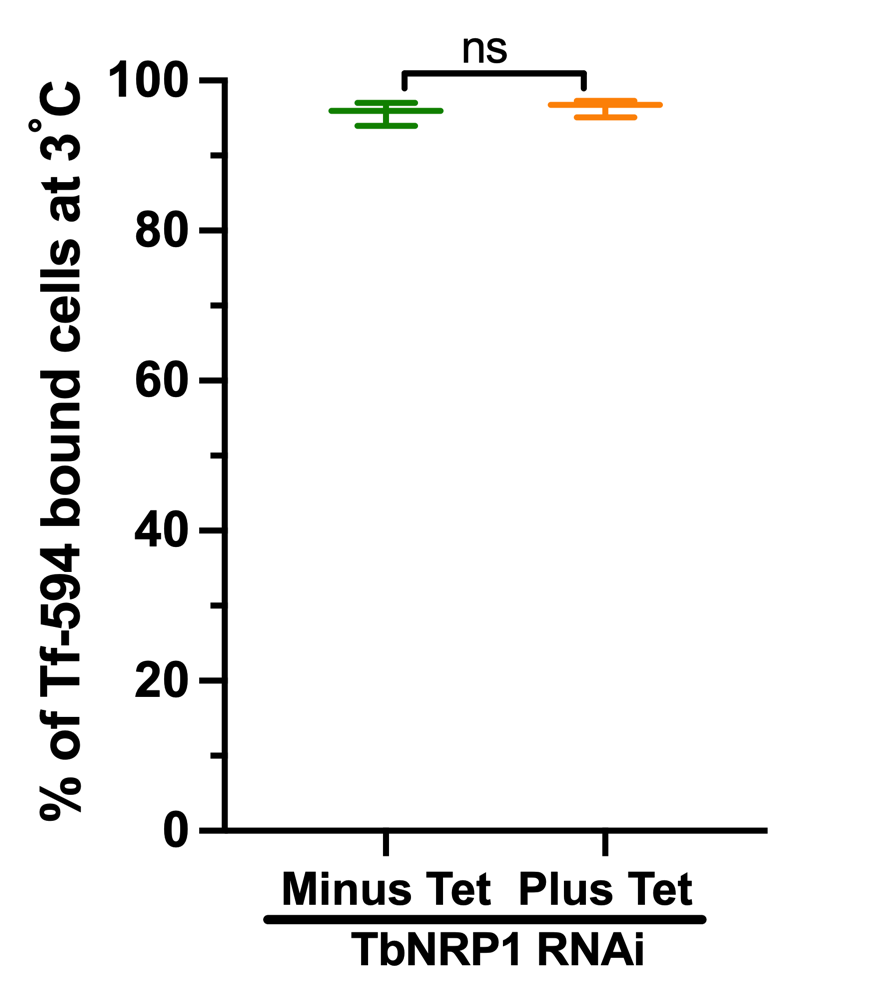


**Supplementary Figure S3.**


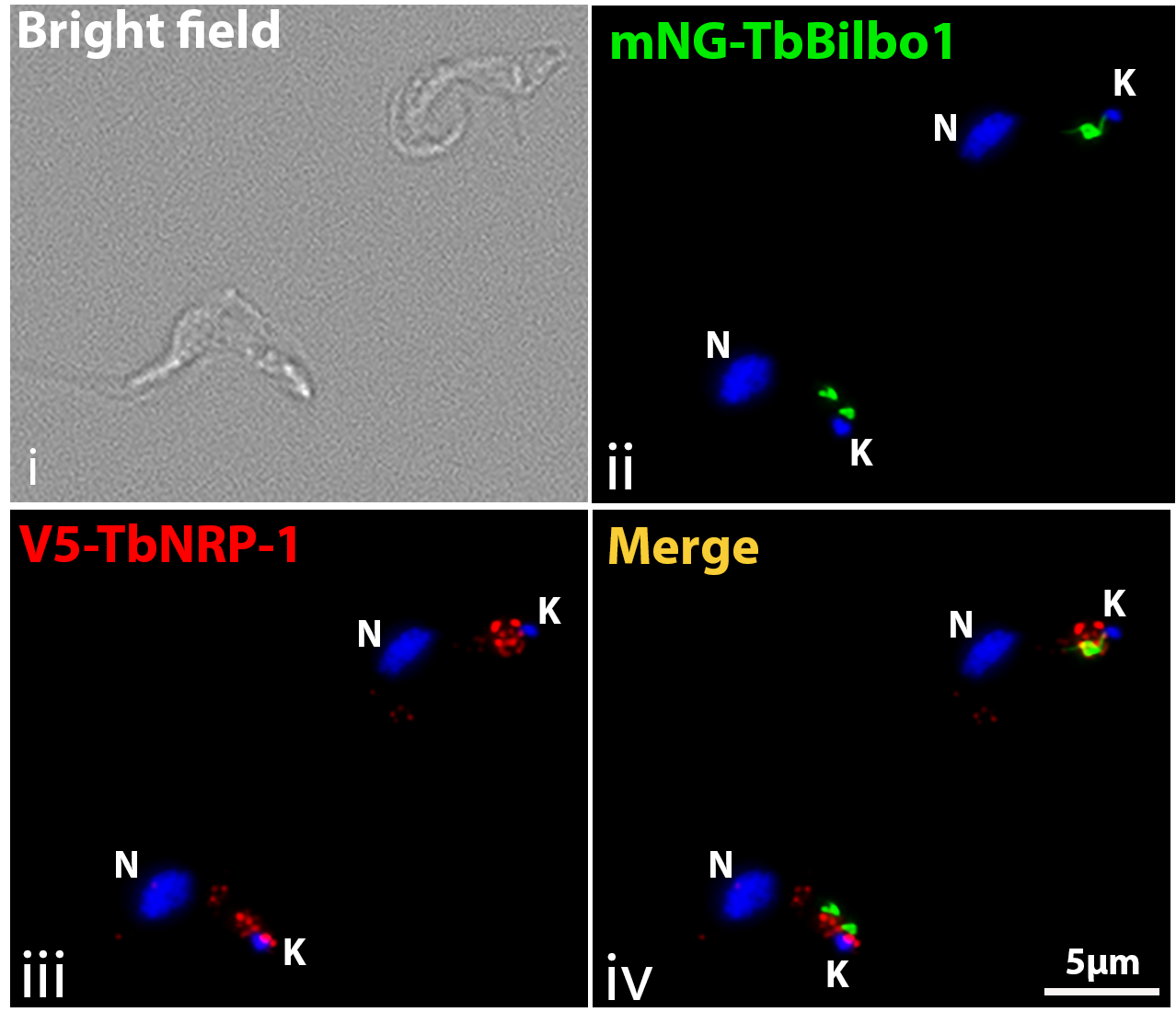


**Supplementary Figure S4.**


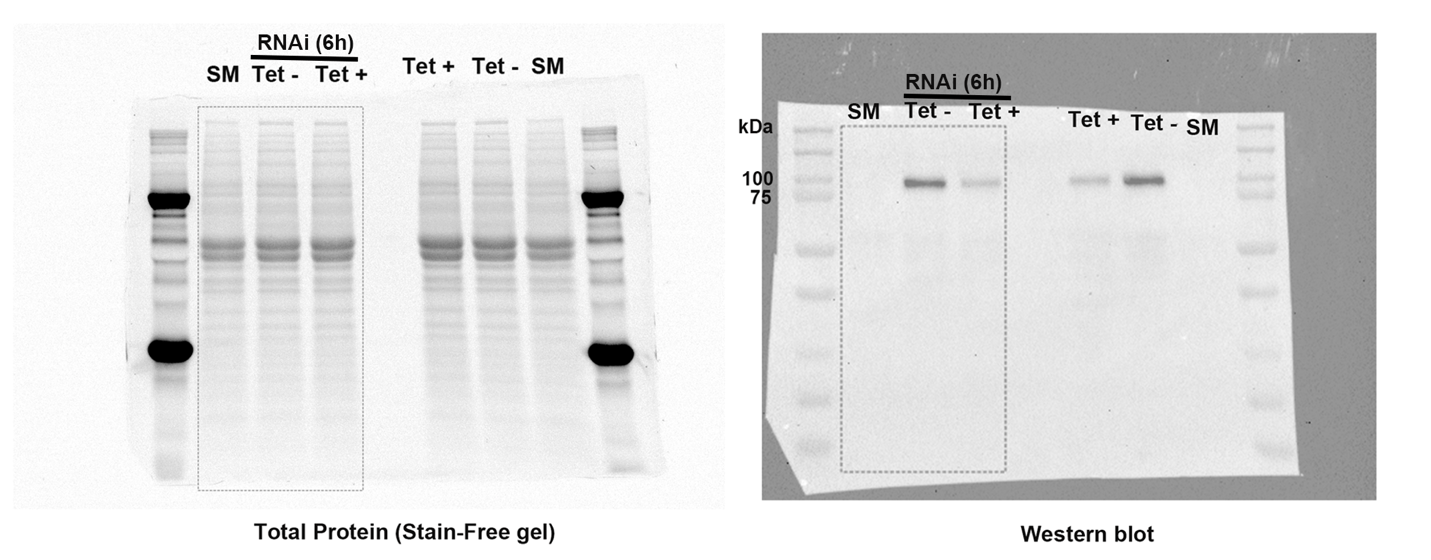


**Supplementary Figure S5.**


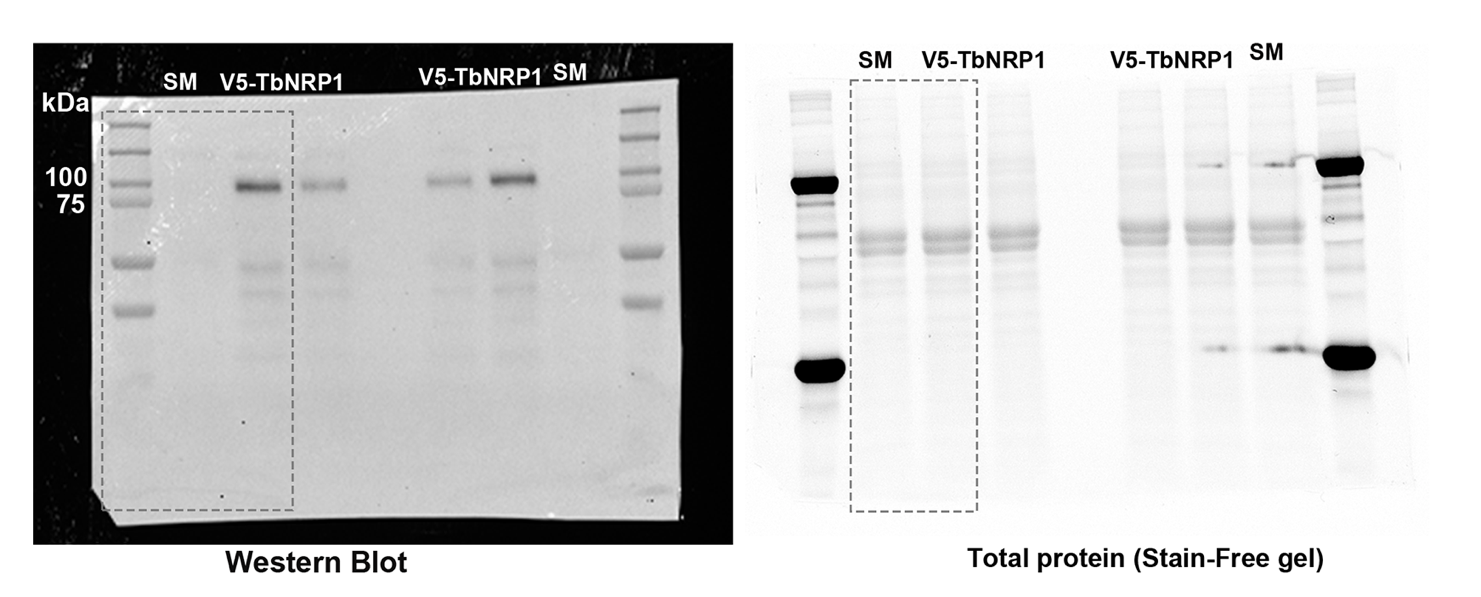


**Supplementary Figure S6.**


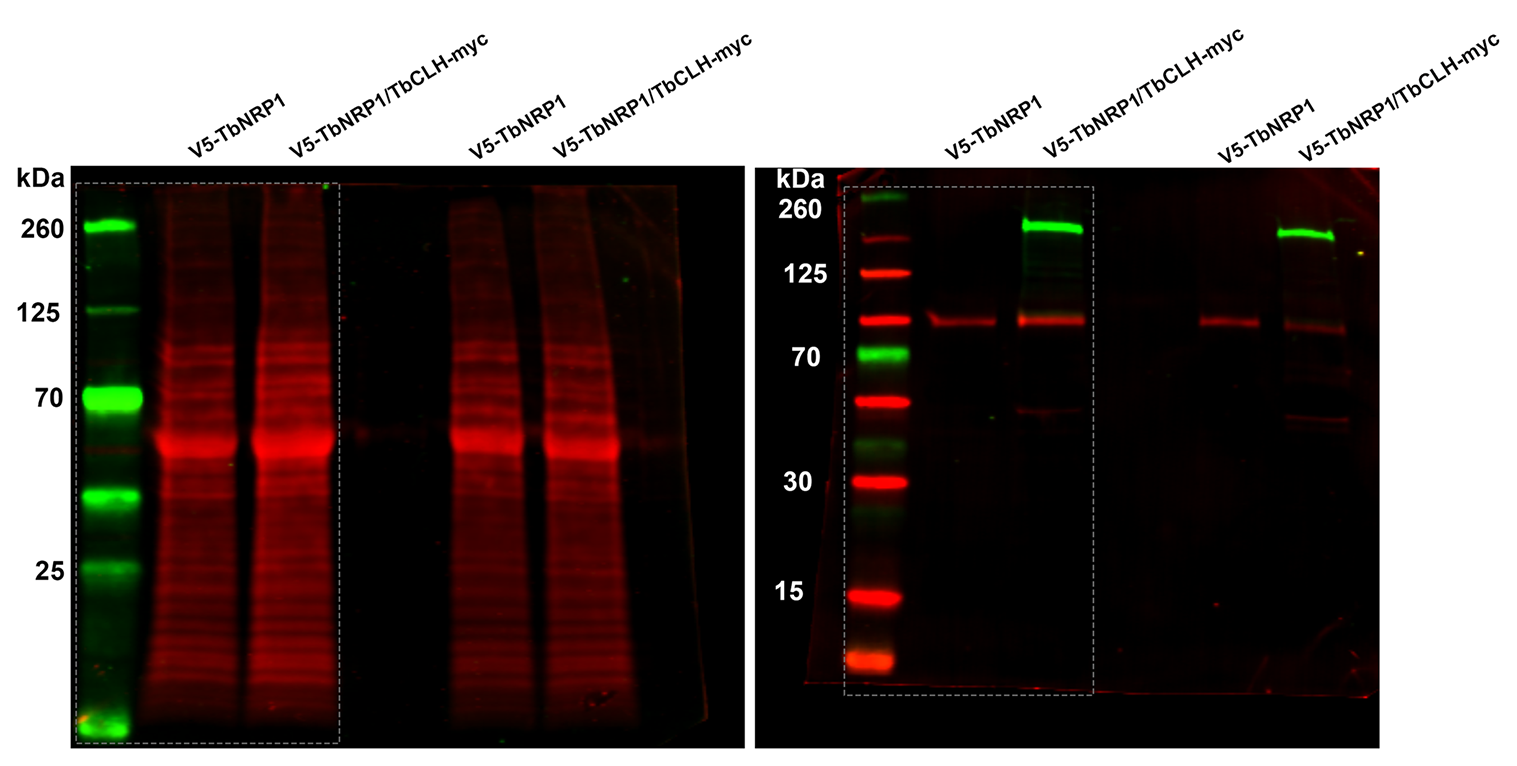


**Supplementary Figure S7.**


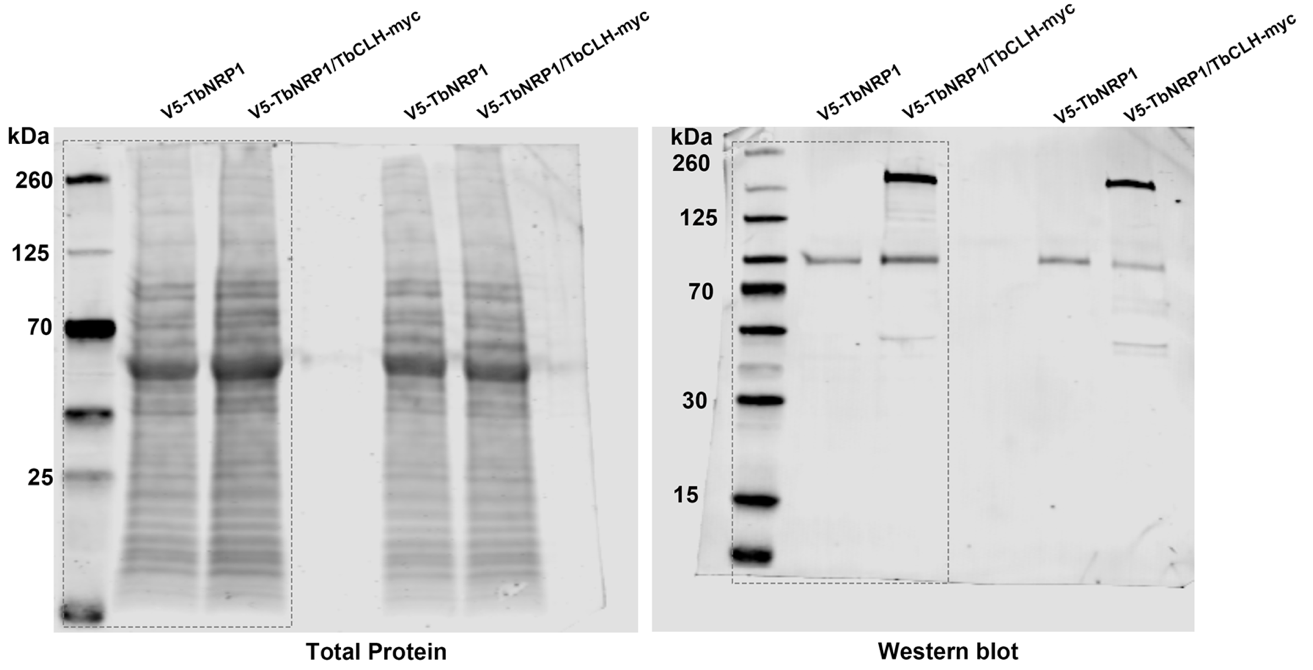

Supplement: Supplementary file 1 — Supplementary Information 1. [file 41598_2022_22054_MOESM1_ESM.docx]
